# Molecular Subtyping in Cholera Outbreak, Laos, 2010

**DOI:** 10.3201/eid1711.110280

**Published:** 2011-11

**Authors:** Noikaseumsy Sithivong, Tomoko Morita-Ishihara, Arounnapha Vongdouangchanh, Traykhouane Phouthavane, Khampheng Chomlasak, Lay Sisavath, Bouaphanh Khamphaphongphane, Bounthanom Sengkeopraseuth, Phengta Vongprachanh, Onechanh Keosavanh, Kongmany Southalack, Lee Jiyoung, Reiko Tsuyuoka, Makoto Ohnishi, Hidemasa Izumiya

**Affiliations:** National Center for Laboratory and Epidemiology, Vientiane, Laos (N. Sithivong, A. Vongdouangchanh, T. Phouthavane, K. Chomlasak, L. Sisavath, B. Khamphaphongphane, B. Sengkeopraseuth, P. Vongprachanh, O. Keosavanh, K. Southalack); National Institute of Infectious Diseases, Tokyo, Japan (T. Morita-Ishihara, L. Jiyoung, M. Ohnishi, H. Izumiya); World Health Organization, Vientiane (R. Tsuyuoka)

**Keywords:** Vibrio infections, cholera, pulsed-field gel electrophoresis, multilocus variable number tandem repeat analysis, bacteria, Laos, dispatch, *Suggested citation for this article:* Sithivong N, Morita-Ishihara T, Vongdouangchanh A, Phouthavane T, Chomlasak K, Sisavath L, et al Molecular subtyping in cholera outbreak, Laos, 2010. Emerg Infect Dis [serial on the Internet]. 2011 Nov [date cited]. http://dx.doi.org/10.3201/eid1711.110280

## Abstract

A cholera outbreak in Laos in July 2010 involved 237 cases, including 4 deaths. Molecular subtyping indicated relatedness between the *Vibrio cholerae* isolates in this and in a 2007 outbreak, uncovering a clonal group of *V. cholerae* circulating in the Mekong basin. Our finding suggests the subtyping methods will affect this relatedness.

Cholera is a major public health concern in countries where access to safe water and adequate sanitation cannot be guaranteed for all residents. *Vibrio cholerae* serogroups O1 and O139 are the causative agents of cholera (*1*). A major virulence factor is cholera toxin (Ctx) encoded by the *ctxAB* gene and located on the Ctx prophage. *V. cholerae* O1 is classified into 2 biotypes, classical and El Tor. The El Tor biotype is responsible for the ongoing seventh pandemic of cholera (*2*). Since the early 1990s, the El Tor variant strains, which are biotypes of El Tor but carry the classical type of *ctxB*, have emerged and prevail in multiple regions where cholera is endemic (*1**,**3**–**6*).

## The Study

In July 2010, a cholera outbreak began in Attapeu Province in southern Laos along the Cambodian border. Onset dates were July 5–September 16. The outbreak spread to 17 villages of the province and involved 237 persons, including 4 who died. To isolate the suspected *V. cholera* colonies, we screened specimens on thiosulfate citrate bile salt sucrose agar with or without enrichment in alkaline peptone water. Suspected colonies were examined by conventional biochemical tests and PCR amplification of *ctx* (*7**,**8*). Of the 42 fecal specimens tested, 9 were culture positive. The isolates were toxigenic *V. cholerae* O1 serotype Ogawa with features of the El Tor variant, according to the *ctxB-*typing method of Morita et al. (*9*).

We analyzed the 9 *V. cholerae* isolates from the Attapeu outbreak. We performed pulsed-field gel electrophoresis (PFGE) according to the PulseNet protocol (*10*) and multilocus variable number tandem repeat analysis (MLVA) using the 7 loci, as described (*5**,**11*).

The isolates of the Attapeu outbreak had almost indistinguishable PFGE profiles and MLVA repeat copy numbers. In PFGE analysis, 8 of the 9 isolates showed indistinguishable profiles (PFGE-A). The profile of the remaining isolate differed from the dominant isolates by 2 bands (PFGE-B) (Figure). In MLVA, 8 isolates showed the same MLVA type (MLVA-I), and 1 isolate showed another MLVA type that differed from the major MLVA type by being a single-locus variant of MLVA-I with only 1 locus and 1 repeat copy number (MLVA-II) (Table). Seven of the MLVA-I and 1 of the MLVA-II isolates showed the PFGE-A profile, and 1 of the MLVA-I isolates showed the PFGE-B profile. Although the source of contamination remains unknown, these results indicate that all isolates were indistinguishable from or similar to each other and that the outbreak could have been caused by a single source of contamination.

**Figure:**
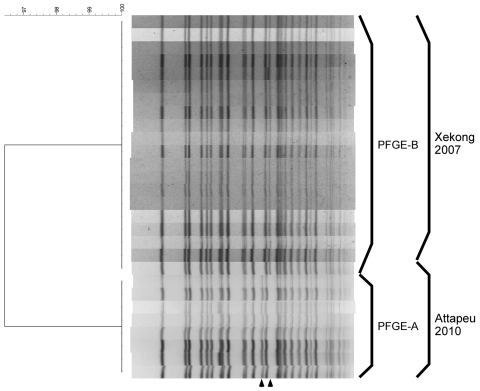
*Not*I-digested pulsed-field gel electrophoresis (PFGE) profiles of *Vibrio cholerae* isolates, Laos, 2010. The names of the profiles and the sources of the isolates are shown on the right. A dendrogram was created with BioNumerics software (Applied Maths, Kortrijk, Belgium) by using the Dice coefficient, unweighted pair-group method with arithmetic means, and a band-position tolerance of 1.2%. Arrowheads at bottom indicate location of bands differing in PFGE-A and PFGE-B.

**Table:** MLVA types identified in study of cholera, Laos, 2010*

MLVA type	No. isolates	Outbreak location	*Vibrio cholerae* repeat copy no.							PFGE profile (no. isolates)
			1	2	3	5	6	7	8	
I	8	Attapeu	8	6	NA	NA	7	17	17	A (7), B (1)
II	1	Attapeu	8	6	NA	NA	7	18	17	A (1)
III	17	Xekong	10	6	NA	4	7	16	16	B (17)
IV	1	Xekong	10	6	NA	4	7	16	17	B (1)
V	1	Xekong	9	6	NA	4	7	16	16	B (1)

*MLVA, multilocus variable number tandem repeat analysis; PFGE, pulsed-field gel electrophoresis; NA, no PCR products amplified.

For comparison, we also examined 19 isolates from an outbreak that occurred in Xekong Province in 2007. These isolates also were toxigenic *V. cholerae* O1 serotype Ogawa of the El Tor variant (*12*). MLVA results clearly indicate that the isolates of the Attapeu outbreak in 2010 differed from those of the Xekong outbreak in 2007. The isolates from the Xekong outbreak comprised 3 MLVA types; 17 isolates were MLVA-III, 1 was MLVA-IV, and 1 was MLVA-V. MLVA-IV and MLVA-V were single-locus variants of MLVA-III (Table). Of the 7 loci tested, 3 or 4 displayed different repeat copy numbers than did those of the Attapeu and Xekong outbreaks. In PFGE analysis, however, the profiles were similar to each other; the isolates from the Xekong outbreak showed a PFGE-B profile (Figure).

These results suggest that strains with a specific PFGE type and the related strains have been circulating in the area for at least 3 years. Nguyen et al. suggested that another cholera outbreak in Vietnam that occurred from the end of 2007 to the beginning of 2008 was associated with the Xekong outbreak (*13*). Choi et al. also studied isolates from Vietnam in 2007 and 2008 by using MLVA, wherein they used 5 loci that are in common with those in this study (VC-1, -2, -6, -7, and -8) (*14*). The MLVA results obtained in our study indicated that the repeat copy numbers of the compatible loci of the Xekong outbreak isolates were the same as those of some of the isolates described in the study by Choi et al. This finding strongly suggests that the causative agents of the Xekong outbreak of Laos and the Vietnam outbreak in 2007–2008 were the same. Moreover, the strains were speculated to circulate widely in the Mekong basin, although the similarity between the PFGE profiles of the isolates from Laos and Vietnam remain to be studied.

Recently, another *ctxB* type of *V. cholerae* O1 biotype El Tor serotype Ogawa was reported in Orissa in eastern India (*15*). Representatives of the Xekong and Attapeu isolates also were subjected to sequence analysis of *ctxB*. The results showed that their *ctxB* sequences were identical with that of the original classical type, which suggests that the clonal group in the Mekong basin differs from the new Orissa type of *V. cholerae* in India.

## Conclusions

Our study clearly indicates that the 2010 cholera outbreak at Attapeu was caused by 1 source of contamination. Furthermore, isolates from the Attapeu outbreak and the 2007 Xekong outbreak showed similar PFGE profiles, but they were differentiated by MLVA, consistent with their origin. This study suggests that PFGE analysis is useful for identifying the kinds of *V. cholerae* clones circulating in a specific geographic region and might be useful for determining a long-term framework of the region-specific *V. cholerae* because PFGE profiles are probably more stable than the MLVA types. By contrast, MLVA is useful for investigating and discriminating short-term individual outbreaks in a region. Another cholera outbreak in Cambodia in 2010 also might be related to the Attapeu outbreak. Combined use of both molecular subtyping methods would indicate the relatedness of cholera in the 2010 Cambodian outbreak and the others in the Mekong basin.
